# Microwave-Enhanced Cross-Coupling Reactions Involving Alkynyltrifluoroborates with Aryl Bromides

**DOI:** 10.3390/molecules18021755

**Published:** 2013-01-29

**Authors:** Vitali Coltuclu, Eric Dadush, Abhijit Naravane, George W. Kabalka

**Affiliations:** Departments of Chemistry and Radiology, University of Tennessee, Knoxville, TN 37996, USA

**Keywords:** organotrifluoroborates, alkynes, microwave, coupling reactions

## Abstract

Palladium-catalyzed alkynylation has emerged as one of the most reliable methods for the synthesis of alkynes which are often used in natural product syntheses and material science. An efficient method for coupling alkynyltrifluoroborates with various aryl bromides in the presence of a palladium catalyst has been developed using microwave irradiation. The microwave reactions are rapid and efficient.

## 1. Introduction

The palladium-catalyzed alkynylation of aromatic rings has emerged as one of the most reliable methods for the synthesis of arylalkynes [1]. Classic methods for preparing aryl alkynes include the copper-promoted Castro-Stephens reaction [2] and the Sonogoshira reaction [3]. Of these, the Sonogoshira reaction is generally considered the most versatile and is used widely in natural product synthesis as well as in materials science [4,5]. Negishi also developed an effective palladium-catalyzed alkynylation reaction utilizing alkynylzinc reagents [6,7] and 1-halo-1-alkynes [8]. In spite of these advances, there are instances in which the alkynyl coupling reactions fail to provide acceptable yields.

The availability of new catalysts and more stable organoborane reagents such as the potassium organotrifluoroborates has broadened the possible applications of alkynyl coupling reactions. Organoboron compounds offer several advantages over other organometallic reagents in that they are generally less toxic and possess remarkable stability when compared to zinc and magnesium reagents [9,10,11,12]. Furthermore, the ease of preparation, stability, and commercial availability of a variety of boron reagents enhance their synthetic utility. At the same time, the stability of many of the new organoboron reagents does not diminish their reactivity in a large variety of reactions [13,14].

We have been exploring the use of microwave irradiation in cross-coupling reactions between potassium organotrifluoroborates and organic halides [15]. Microwaves have become increasingly important in organic syntheses in that minimal amounts of solvent are required and reactions often proceed under catalyst-free conditions. We recently reported the use of microwaves for cross-coupling reactions involving both alkynyltrifluoroborates and vinyltrifluoroborates [16]. In that study, moderate yields of the desired coupled products were obtained when aryl iodides were coupled with alkynyltrifluoroborates. In a continuation of those studies, we have since discovered that the reactions are much more efficient when aryl bromides are utilized as the coupling partners. We wish to report the results of a study focused on the cross-coupling reactions of various potassium alkynyltrifluoroborates with aryl bromide reagents using microwave irradiation.

## 2. Results and Discussion

Molander and co-workers reported the successful coupling of aryl bromides with alkynyltrifluoroborates under thermal conditions [17]. Their syntheses generally requires long reaction times (12 h) and relatively high catalyst loadings (9 mol%). Using microwave irradiation, we have found that the reactions can be carried out in 20 min in the presence less catalyst. Typical reaction conditions involve a catalyst loading of 2 mol% PdCl_2_(dppf)-CH_2_Cl_2_ while providing yields comparable to the thermal reactions.

We initiated the current study by coupling a variety of substituted aryl bromides with potassium 1-octynyltrifluoroborate (Table 1). The presence of an electron-withdrawing group on the aryl bromide slightly enhances the yield of the desired coupling reaction (*i.e.*, a cyano group). The presence of an additional halide (for example, chlorine in 4-bromochlorobenzene) does not affect the reaction yield but demonstrates that bromine is more reactive than chlorine in the coupling reaction. The presence of an electron-donating substituent, such as a methyl or methoxy group, leads to lower yields. Increasing the reaction time also results in lower yields due to decomposition reactions.

4-Bromobenzonitrile was then coupled with various potassium alkynyltrifluoroborates (Table 2). The reaction works well with essentially all of the alkynyltrifluoroborate reagents investigated. However, the presence of a free hydroxyl group is detrimental to the coupling reaction; since the starting 6-hydroxy-1-hexynyltrifluoroborate is consumed in the reaction, it is possible that protonolysis of the carbon-boron bond is occurring. Further investigations are underway. The somewhat lower yield obtained when using the 2-(4-cyanophenyl)ethynyltrifluoroborate (entry 9) is also puzzling since the corresponding 4-methoxy derivative gave an excellent yield of the desired product.

## 3. Experimental

### 3.1. General Information

The palladium catalyst, PdCl_2_(dppf)•CH_2_Cl_2_ and most of the potassium trifluoroborate reagents were supplied by Frontier Scientific Inc. (Logan, UT, USA) and were used as received. Potassium(2-methyl-1-buten-3-yn-4-yl)trifluoroborate, potassium (1-octyn-1-yl)trifluoroborate, potassium [1-(3,3-dimethyl)-butyn-1-yl]trifluoroborate, and potassium (4-methoxyphenylethynyl)trifluoroborate were prepared according to published procedures [17,18,19]. *N,N*-Diisopropylethylamine and the aryl bromides were purchased from Sigma-Aldrich (St. Louis, MO, USA) and used without further purification. Column chromatography was performed using silica gel (60 Å, 230–400 mesh, ICN Biomedicals GmbH, Eschwege, Germany). Analytical thin-layer chromatography was performed using 250 μm silica plates (Analtech, Inc., Newark, DE, USA). ^1^H-NMR and ^13^C-NMR spectra were recorded at 400 and 101 MHz, respectively, on a Bruker Avance 400 instrument. Chemical shifts are referenced to TMS and measured with respect to the residual protons in the deuterated solvents.

### 3.2. Coupling Reactions: General Procedure

To a dry Pyrex tube containing a magnetic stir bar, was added a potassium organotrifluoroborate (0.50 mmol) and PdCl_2_(dppf)•CH_2_Cl_2_ (0.01 mmol). The tube was then capped with an airtight rubber cap and flushed with argon to maintain a moisture and oxygen free environment. The aryl bromide (0.50 mmol) and Hünig’s base (265 µL) were then added via syringe (in our hands, Hünig’s base provided more consistent results than carbonate salts). A mixture of 2-propanol/water (2:1, 5.0 mL) was added to the tube, followed by an argon purge. The resulting mixture was placed in a CEM microwave unit in the closed vessel mode and allowed to react at 100 °C for 20 min. The reaction mixture was then transferred to a separatory funnel and diluted with diethyl ether (20 mL). The mixture was washed with water (3 × 20 mL) to remove byproducts. The organic layer was separated, dried over anhydrous sodium sulfate, filtered, concentrated, and the product isolated by silica gel chromatography using hexane/ethyl acetate (100/1) as eluent. The solvents were removed using a rotary evaporator to yield pure product. NMR spectra were compared to the previously reported data.

*4-(1-Octynyl)benzonitrile*. Yellow oil. ^1^H-NMR (CDCl_3_): δ 7.56 (d, *J* = 8.4 Hz, 2H), 7.45 (d, *J* = 8.6 Hz, 2H), 2.42 (t, *J* = 7.2 Hz, 2H), 1.67 (t, *J* = 7.4 Hz, 2H), 1.51–1.42 (m, 2H), 1.40–1.27 (m, 4H), 0.92 (t, *J* = 7.4 Hz, 3H). ^13^C-NMR (CDCl_3_): δ 132.4, 132.2, 129.5, 118.9, 111.2, 96.0, 79.8, 31.7, 28.9, 28.8, 22.9, 19.9, 14.4.

*1-(4-Chlorophenyl)-1-octyne*. Yellow oil. ^1^H-NMR (CDCl_3_): 7.31 (d, *J* = 8.5 Hz, 2H), 7.24 (d, *J* = 8.6 Hz, 2H), 2.38 (t, *J* = 7.1 Hz, 2H), 1.63–1.55 (m, 2H), 1.48–1.40 (m, 2H), 1.35–1.30 (m, 4H), 0.90 (t, *J* = 6.6 Hz, 3H). ^13^C-NMR (CDCl_3_): δ 133.8, 133.2, 128.9, 123.1, 92.0, 80.0, 31.8, 29.1, 29.1, 23.0, 19.9, 14.5.

*1-(4-Methoxyphenyl)-1-octyne*. Yellow oil. ^1^H-NMR (CDCl_3_): δ 7.32 (d, *J* = 8.6 Hz, 2H), 6.80 (d, *J* = 8.6 Hz, 2H), 3.78 (s, 3H), 2.38 (t, *J* = 7.2 Hz, 2H), 1.62–1.54 (m, 2H), 1.50–1.44 (m, 2H), 1.34–1.32 (m, 4H), 0.90 (t, *J* = 6.8 Hz, 3H). ^13^C-NMR (CDCl_3_): δ 159.5, 133.3, 116.8, 114.3, 89.3, 80.7, 55.7, 31.9, 29.3, 29.0, 23.0, 19.9, 14.5.

*1-(3-Methoxyphenyl)-1-octyne*. Yellow oil. ^1^H-NMR (CDCl_3_): δ 7.17 (t, *J* = 7.9 Hz, 1H), 6.98 (d, *J* = 7.6 Hz, 1H), 6.81 (dd, *J* = 8.2, 2.8 Hz, 1H), 3.77 (s, 3H), 2.39 (t, *J* = 7.0 Hz, 2H), 1.59 (m, *J* = 13.2, 6.3, 5.7 Hz, 2H), 1.46 (m, *J* = 8.7, 8.0 Hz, 3H), 1.32 (m, *J* = 8.8, 4.6 Hz, 4H), 0.90 (t, *J* = 6.0 Hz, 3H). ^13^C-NMR (CDCl_3_): δ 159.8, 129.6, 125.6, 124.6, 116.9, 114.5, 90.8, 81.0, 55.6, 31.8, 29.2, 29.1, 23.0, 19.9, 14.5.

*1-(4-Methylphenyl)-1-octyne*. Yellow oil. ^1^H-NMR (CDCl_3_): δ 7.30 (d, *J* = 7.9 Hz, 2H), 7.10 (d, *J* = 7.9 Hz, 2H), 2.38 (t, *J* = 7.0 Hz, 2H), 2.31 (s, 3H), 1.66–1.51 (m, 2H), 1.52–1.43 (m, 2H), 1.38–1.25 (m, 4H), 0.90 (s, *J* = 6.7 Hz, 3H). ^13^C-NMR (CDCl_3_): δ 137.9, 132.1, 129.5, 121.9, 90.3, 80.9, 31.8, 29.4, 28.8, 22.8, 21.6, 19.6, 14.9.

*4-(6-Chlorohex-1-yn-1-yl)benzonitrile*. Yellow oil. ^1^H-NMR (CDCl_3_): δ 7.57 (d, *J* = 8.3 Hz, 2H), 7.45 (d, *J* = 8.3 Hz, 2H), 3.60 (t, *J* = 6.5 Hz, 2H), 2.49 (t, *J* = 6.9 Hz, 2H), 1.95 (p, *J* = 6.7 Hz, 2H), 1.78 (p, *J* = 7.1 Hz, 2H). ^13^C-NMR (CDCl_3_): δ 132.5, 132.3, 129.2, 118.9, 111.4, 94.8, 80.4, 44.8, 32.0, 26.0, 19.2.

*4-(3-Methylbut-3-en-1-yn-1-yl)benzonitrile*. White solid. ^1^H-NMR (CDCl_3_): δ 7.52 (dd, *J* = 6.8, 1.9 Hz, 2H), 7.47 (dd, *J* = 6.6, 1.7 Hz, 2H), 5.42 (q, *J* = 0.8 Hz, 1H), 5.39–5.35 (m, 1H), 2.01 (t, *J* = 1.2 Hz, 3H). ^13^C-NMR (CDCl_3_): δ 131.3, 131.1, 128.0, 126.3, 123.1, 119.2, 111.9, 95.1, 86.5, 23.1.

*4-(4-tert-Butyldimethylsiloxy-1-butyn-1-yl)benzonitrile.* Yellow oil. ^1^H-NMR (CDCl_3_): δ 55 (d, *J* = 8.4 Hz, 2H), 7.44 (d, *J* = 8.5 Hz, 2H), 3.81 (t, *J* = 6.8 Hz, 2H), 2.64 (t, *J* = 6.8 Hz, 2H), 0.91 (s, 9H), 0.09 (s, 6H). ^13^C-NMR (CDCl_3_): δ 132.5, 132.3, 129.3, 119.0, 111.5, 93.0, 80.7, 61.9, 26.3, 24.4, 18.7, −4.8.

*4-(3,3-Dimethylbut-1-ynyl)-benzonitrile*. White solid. ^1^H-NMR (CDCl_3_): δ 8.48 (d, *J* = 8 Hz, 2H), 7.42 (d, *J* = 8 Hz, 2H), 1.35 (s, 9H). ^13^C-NMR (CDCl_3_): δ 132.7, 132.5, 129.7, 120.0, 111.2, 103.9, 78.8, 31.3, 28.6.

*4-((Trimethylsilyl)ethynyl)benzonitrile*. Light yellow solid. ^1^H-NMR (CDCl_3_): δ 7.56 (d, *J* = 8.5 Hz, 2H), 7.50 (d, *J* = 8.5 Hz, 2H), 0.27 (s, 9H). ^13^C-NMR (CDCl_3_): δ 133.1, 132.2, 127.9, 118.5, 112.0, 103.1, 100.0, −0.5.

*4-Phenylethynylbenzonitrile*. White solid. ^1^H-NMR (CDCl_3_): δ 7.62 (m, 4H), 7.55 (m, 2H), 7.36 (m, 3H). ^13^C-NMR (CDCl_3_): δ 132.3, 132.1, 131.7, 129.2, 128.5, 128.3, 122.3, 118.6, 111.6, 93.9, 87.8.

*4-((4-Methoxyphenyl)ethynyl)benzonitrile*. White solid. ^1^H-NMR (CDCl_3_): δ 7.59 (q, *J* = 8.5 Hz, 4H), 7.48 (d, *J* = 8.8 Hz, 2H), 6.90 (d, *J* = 8.8 Hz, 2H), 3.84 (s, 3H). ^13^C-NMR (CDCl_3_): δ 160.8, 133.8, 132.5, 132.3, 129.1, 119.1, 114.8, 114.7, 111.5, 94.6, 87.2, 55.8.

*4,4′-Dicyanophenylethynylene*. White solid. ^1^H-NMR (CDCl_3_): δ 7.66 (4H, d, *J* = 8.2 Hz), 7.64 (4H, d, *J* = 8.2Hz). ^13^C-NMR (CDCl_3_): δ 132.3, 132.2, 127.1, 118.5, 112.6, 91.9.

## 5. Conclusions

An efficient method for coupling alkynyltrifluoroborates with various aryl bromides in the presence of a palladium catalyst has been developed using microwave irradiation. The reaction is straightforward and rapid, producing the coupled products in good to excellent yields.

## Figures and Tables

**Scheme 1:**
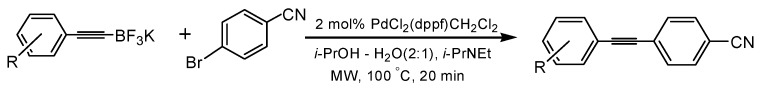
Cross-coupling reaction of alkynyltrifluoroborates.

**Table 1:** Cross-coupling of aryl bromides with potassium octynyltrifluroborate ^a^.

Entry	R^1^BF_3_K	Ar-Br	Ar-R^1^	Yield (%) ^b^
1	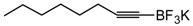	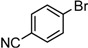	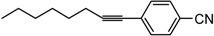	75
2	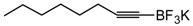	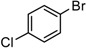	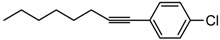	65
3	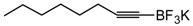	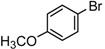	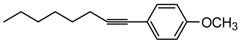	60
4	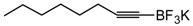	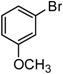	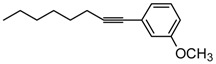	54
5	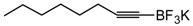	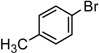	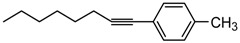	40

^a^ All reactions carried out at 100 °C; ^b^ Yields of pure products isolated by silica gel chromatography.

**Table 2:** Cross-coupling reactions of alkynyltrifluoroborates with 4-bromobenzonitrile ^a^.

Entry	R^1^BF_3_K	Ar-Br	Ar-R^1^	Yield (%) ^b^
1	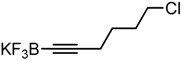	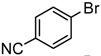	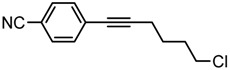	84
2	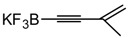	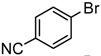	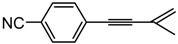	87
3	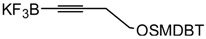	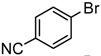	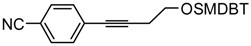	87
4	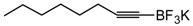	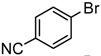	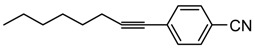	75
5	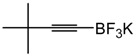	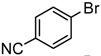	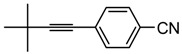	80
6	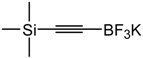	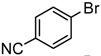	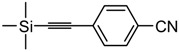	76
7	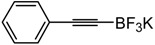	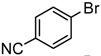	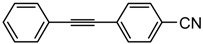	70
8	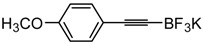	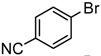	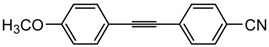	78
9	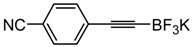	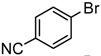	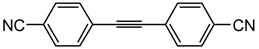	45
10	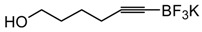	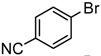	NR	

^a^ All reactions were carried out at 100 **°**C; ^b^ All yields are of pure products isolated by silica gel chromatography.
